# Scoring Alternatives for Mental Speed Tests: Measurement Issues and Validity for Working Memory Capacity and the Attentional Blink Effect

**DOI:** 10.3390/jintelligence6040047

**Published:** 2018-10-15

**Authors:** Florian Schmitz, Dominik Rotter, Oliver Wilhelm

**Affiliations:** Institute of Psychology, Ulm University, Albert-Einstein Allee 47, 89081 Ulm, Germany; dominik.rotter@uni-ulm.de (D.R.); oliver.wilhelm@uni-ulm.de (O.W.)

**Keywords:** mental speed, working memory capacity, attentional blink, response time modeling

## Abstract

Research suggests that the relation of mental speed with working memory capacity (WMC) depends on complexity and scoring methods of speed tasks and the type of task used to assess capacity limits in working memory. In the present study, we included conventional binding/updating measures of WMC as well as rapid serial visual presentation paradigms. The latter allowed for a computation of the attentional blink (AB) effect that was argued to measure capacity limitations at the encoding stage of working memory. Mental speed was assessed with a set of tasks and scored by diverse methods, including response time (RT) based scores, as well as ex-Gaussian and diffusion model parameterization. Relations of latent factors were investigated using structure equation modeling techniques. RT-based scores of mental speed yielded substantial correlations with WMC but only weak relations with the AB effect, while WMC and the AB magnitude were independent. The strength of the speed-WMC relation was shown to depend on task type. Additionally, the increase in predictive validity across RT quantiles changed across task types, suggesting that the worst performance rule (WPR) depends on task characteristics. In contrast to the latter, relations of speed with the AB effect did not change across RT quantiles. Relations of the model parameters were consistently found for the ex-Gaussian tau parameter and the diffusion model drift rate. However, depending on task type, other parameters showed plausible relations as well. The finding that characteristics of mental speed tasks determined the overall strength of relations with WMC, the occurrence of a WPR effect, and the specific pattern of relations of model parameters, implies that mental speed tasks are not exchangeable measurement tools. In spite of reflecting a general factor of mental speed, different speed tasks possess different requirements, supporting the notion of mental speed as a hierarchical construct.

## 1. Introduction

### 1.1. Mental Speed and Its Relation with Intelligence

A classical definition of mental speed tasks is that they are simple enough so that anyone can solve them given sufficient time [1]. According to Kyllonen and Christal [2], a broad range of elementary processes can be subsumed under this definition. This comprises the encoding of stimuli into working memory as well as the recall of information stored in long-term memory, even the execution of a motor response. Consequently, a multitude of tasks qualifies as measures of mental speed, as the construct has been conceptualized as multi-faceted and hierarchical, comprising several group factors [3]. This has been confirmed both for paper-and-pencil tests of mental speed [4] as well as for computerized equivalents [5]. However, one central aspect pertains to almost all measures of mental speed: They are moderately correlated with measures of general intelligence. According to a reductionist perspective, mental speed is correlated with intelligence, because speed of processing causally contributes to higher cognitive processes [6], e.g., by constituting a good neural efficacy see also [7]. Other theoretical accounts postulate working memory capacity (WMC) as the major basis of fluid intelligence and reasoning ability [2,8,9,10]. However, it has been argued, too, that mental speed contributes to WMC, as processing and rehearsal rely on a shared, time-dependent resource [11]. In contrast to the former, not all authors conceive mental speed as a causal basis of intelligence or WMC. In line with hierarchical conceptualizations of mental abilities, it was argued instead that mental speed is one important group factor among others [12].

Whatever the reason, mental speed is moderately correlated with intelligence, which has been demonstrated in large scale population-based studies [13] and meta analyses [14,15]. However, a number of moderating factors have been identified. For instance, it is a well-known finding that task complexity moderates the validity of mental speed tasks predicting intelligence [16,17]. The more complex the task, e.g., in terms of response alternatives; [17], the slower are response times (RT) on average, and the higher the correlation with measures of intelligence [14,15].

After focusing almost exclusively on mean response times for a long time, research conducted in the last decades has addressed alternative performance indicators derived from speed tasks. It has been postulated that inter-trial variability (*SD*_RT_) would be more strongly related with intelligence than mean RT (*M*_RT_) [18]. This notion builds on the assumption that increased variability of the response times indicates an impaired functioning of their underlying mental process. This prediction can be reconciled with the finding that the developmental trajectory of *SD*_RT_ resembles that of cognitive ability. *SD*_RT_ has been shown [19] to be lowest in young adults whose cognitive functioning is high, whereas higher *SD*_RT_ have been reported for both younger children and older adults whose cognitive functioning is lower relative to that of young adults. A recent meta analysis conducted across 27 independent samples has confirmed that response time variability is moderately related with intelligence, however, not consistently more highly than mean response times [14]. Additionally, *M*_RT_ and *SD*_RT_ have been shown to be highly collinear (*r* ≈ 0.90) across participants [20]. In part, slow extreme values may contribute to this correlation, as they affect both mean and variability of responding in the speed tasks. Additionally, they may contribute to the relations of both metrics with intelligence. In support of this notion, it has been shown that the mean RT (*M*_RT_) is more strongly related with intelligence than the median RT (*Mdn*_RT_) [21,22], where the former is affected but not the latter is affected by slow extreme values.

Further evidence for the validity of slow response times has been obtained in a classical study conducted by Larson and Alderton [23]. They divided responses times into a number of RT bands, and found that validity for intelligence has been the higher the slower the response band. This finding constituted the worst performance rule (WPR) which has been confirmed in many studies see [24] for a review. Building on these findings, the differential development model [25] postulates that besides the general speed of processing, lapses of attention mediate the relationship of age with intelligence.

However, the WPR has not been supported in all studies, and some research has revealed that all portions of the RT distribution contain comparable validity for assessing individual differences in ability [26,27]. It has been suggested that the WPR is moderated by task complexity, as it should rather occur in complex tasks compared to simpler ones [28]. In fact, recent research using experimental manipulations of task complexity in speed tasks confirmed this assumption. It has been demonstrated that validity of slow responses increased with complexity of the speed task [29], and it has been ruled likely that WMC requirements in the speed tasks are responsible for the increase in validity for intelligence [30].

### 1.2. Modeling of Response Times

Typically observed RT distributions are one-peak functions that are heavily skewed to the right, i.e., they possess a heavy tail to the right side with slow responses. A number of statistical models have been suggested that can be used to approximate the empirically observed shape of the RT distribution. The ex-Gaussian parameterization is a two-component model of response times resulting from folding a Gaussian normal distribution with an exponential distribution. Therefore, it yields three parameters, *μ* and *σ* for the mean and the standard deviation of the Gaussian normal component, and *τ* as the parameter (mean and standard deviation) of the exponential component. The ex-Gaussian model has been shown to be useful in RT research [31,32,33]. The *μ* parameter can be used as a bias-corrected estimate of the mean RT, whereas *σ* corresponds with the general variability of the response times. In contrast, the *τ* parameter characterizes the skew of the distribution, and is, hence, sensitive to the proportion of slow extreme values.

In spite of the model’s descriptive nature, some authors have used parameter estimates to infer theoretically postulated processes. Although there is no consensus which processes are captured by the ex-Gaussian parameters, most authors agree that *τ* is the most relevant parameter for cognitive functioning. Traditionally, the exponential component has been argued to represent the actual decision process, whereas the Gaussian component reflects the non-decision component [34]. Others have argued that the exponential component, which corresponds to the proportion of slow RT values, indexes lapses of attention, and other interruptions in cognitive processes [35,36]. In turn, the Gaussian component has been suggested to subsume multiple sources of noise during stimulus processing, or to correspond with motor processes [37].

In line with these predictions, most studies have confirmed that the ex-Gaussian *τ* parameter is consistently related with intelligence and working memory capacity [27,30,38,39], at least when modeling RT distributions obtained with adults performing elementary cognitive tasks. In contrast to these findings, a recent study conducted with children and adolescents that completed arguably simple RT tasks [40] revealed a significant relation only for the Gaussian *σ* parameter.

Using its three parameters the ex-Gaussian model offers richer information than most conventional RT scores. However, like most other RT scores, only correct responses are considered. Additionally no information is provided concerning the accuracy of responding, so a possible speed-accuracy trade-off cannot be detected.

**Figure 1:**
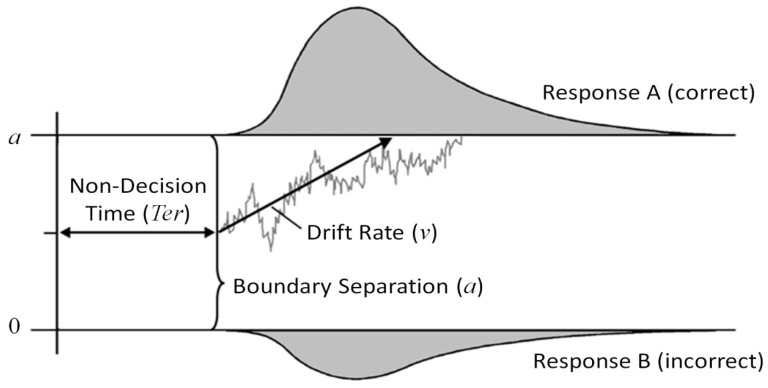
Diffusion model for binary decision tasks.

The diffusion model [41,42] can better cope with these challenges. Encompassing a parsimonious account of a binary decision process, the model allows for a dissociation of the efficacy of information processing from the setting of the speed-accuracy compromise, among others. Specifically, the model conceptualizes a binary decision as a random walk process which originates from a starting point between two response thresholds corresponding to the two response options (see Figure 1). Systematic information and random noise contribute to fluctuation of the decision process across time (see the gray line as a hypothetical sample path in one trial). As soon as the process hits one of the decision thresholds (0 or *a*), the corresponding response is elicited. The mean slope of the decision process, i.e., the rate of evidence accumulation across time, is called the drift rate (*ν*), and was shown to reflect task easiness [43] or ability of the participant [38,39,40,44]. The distance of the two response thresholds denotes the response criterion (*a*). Large distances correspond with cautious information processing [41]: The risk that random fluctuations of the decision process accidently hit the wrong threshold is small, however, the decision will take more time to reach the correct response threshold on average. Consequently, the setting of the response criterion corresponds with the speed-accuracy trade-off. Additionally, a non-decision parameter (*T*_er_) captures time for processes outside the actual decision phase, such as encoding of stimuli or executing motor responses. The full diffusion model [42] comprises additional parameters such as a bias in starting point towards one of the response options, and the inter-trial variabilities of all afore-mentioned parameters.

The diffusion model has been suggested as a theoretical explanation for a number of replicated findings in RT research. Using simulations, it has been demonstrated that variation in the diffusion model’s parameters can account for the typical right-skewed shape of the RT distribution, for the theoretically predicted higher validity of *SD*_RT_ over *M*_RT_, the higher validity of *M*_RT_ over *Mdn*_RT_, and the linear correlation of *M*_RT_ and *SD*_RT_, among others [45,46]. Interestingly, these phenomena can be parsimoniously accounted for by individual differences in the diffusion model’s drift rate that is also responsible for the relation of these observed effects with intelligence. It has to be discussed, though, that the theoretically predicted superiority of *SD*_RT_ over *M*_RT_ has not been approved meta-analytically on the basis of empirical studies [14], and the superiority of the *M*_RT_ over *Mdn*_RT_ shown in some studies [21,22] has been found to be rather marginal in others [39].

As the diffusion model offers a theoretical explanation of the observed shape of the RT distribution, it has been argued that the diffusion model should be preferred to the more descriptive ex-Gaussian parameterization [34,38]. That said, a combination of diffusion model parameters can explain findings in ex-Gaussian parameters. However, there is no one-to-one correspondence of the parameters in both models. In fact, their correlations have been shown to be moderate [34,38,47], even when all relations are estimated as correlations between latent factors [39], thereby removing the problem of insufficient reliability of the parameter estimates. This suggests that parameters estimated in both models possess specificity that could lead to differential validity of specific processes and abilities. However, this has to be demonstrated empirically.

For the purpose of individual differences research, it is a vital question whether model-based parameters can be conceived as “measures of trait-like cognitive styles and abilities” [48] (p. 4). Relevant prerequisites to this end include sufficiently high temporal stability and a certain breadth across a set of different tasks. In fact, temporal stability across a period of eight months has been demonstrated for the most relevant diffusion model parameters, i.e., for the drift rate, for the response criterion, and for the non-decision parameter [49]. However, research has also shown that parameters modeled in different tasks possess considerable specificity [39,49]. Hence, the only moderate correlations between parameters do not result only from insufficient reliability of the estimates, but also from task specificity. Of course, this issue is not confined to modeling approaches, and confirms the hierarchical nature of the speed construct [3,4,5].

However, a particular challenge when fitting models with several parameters concurrently to data is to cope with computational dependencies. This means, an over- or underestimation of one parameter can be compensated by another parameter, resulting in a comparable model fit. This complicates the identification of the true parameters and can, occasionally, result in relatively extreme parameter estimates that are detrimental in correlative research. Such trade-offs have been demonstrated for the ex-Gaussian model [38,47] and for the diffusion model [50]. In their influential study, Schmiedek et al. [38] suggested structural equation modeling to overcome this problem. Potentially error-prone parameter estimates obtained from several indicator tasks are treated as observed variables in a confirmatory factor analyses. Parameters of the same kind (e.g., drift rates) are specified as loadings on a common factor that accounts for their communality. In turn, these factors can be used to test the validity of the “true” parameter variances. Meanwhile, the method of combining parameter estimates with structure equation modeling was successfully applied in a couple of studies [39,40].

Finally, we would like to discuss the possibility of differential parameter validity when complexity of speed tasks varies. Classical research shows that speed tasks discriminate better between persons of different ability when complexity is increased [16,17]. Given that ability is primarily reflected in the diffusions model’s drift rate [38], a number of drift rate associated effects [45] can be predicted to covary more strongly with intelligence at higher task difficulty, including the position of the RT distribution, its skew, and its proportion of slow extreme values.

Accordingly, task complexity can be expected to moderate the validity of the ex-Gaussian parameters for ability, although differentially [20], cf. [45]. When tasks are very simple, individual RT distributions can be expected to largely overlap, and only the *τ* parameter would reliably discriminate between persons of different ability. With increasing task complexity, these differences would become even larger and possibly gain validity. Additionally, the location of the RT distributions would vary more strongly at high task difficulty. Consequently, the *μ* parameter would reflect individual differences more reliably and would possibly gain validity with increasing task difficulty. Additionally, both location (*μ*) and skew (*τ*) would contribute to better discrimination of slow response bands with increasing task complexity. This has been confirmed in previous research [28,29].

Only limited research has addressed differential validity of model parameters across levels of task complexity. Indirect evidence comes from a study [29] in which complexity was manipulated in a Hick paradigm using 0, 1, and 2-bit conditions. Correlations with intelligence were computed for 6 RT bands, and were found to increase across RT bands in all complexity conditions, confirming the WPR [23,24]. However, the increase across RT bands was steepest in the simplest 0-bit condition. Correlations were generally much stronger in the most complex 2-bit condition, but the increase across RT bands was attenuated. From an ex-Gaussian perspective, this pattern suggest that the skew of the RT distribution (corresponding with *τ*) is most predictive of intelligence in simple tasks (in relative terms), whereas the mean (corresponding with *μ*) that contributes to the position of all RT bands gains predictive validity with increasing task complexity. There is only limited direct evidence for differential parameter validity across levels of task complexity. Two studies reported stronger relations with intelligence for the *τ* parameter at higher task complexity, at least descriptively [30,39]. However, results were mixed for the *μ* parameter, either showing the predicted increase [39] or a decrease [30]. In one of these studies [39], results were reported as well for the diffusion model. Relations with intelligence and working memory capacity were parsimoniously accounted for by variation in drift rate, and these relations were slightly stronger at higher task complexity.

### 1.3. Mental Speed, WMC, and the Attentional Blink Effect

Intelligence tests and WMC paradigms have been extensively employed as criterion variables in previous research addressing the validity of alternative speed scores and modeling approaches [30,38,39,40]. Also the current study comprised a set of WMC paradigms. This was motivated by the finding that mental speed and WMC are correlated in a mean magnitude [38,39], so that range restrictions in either direction are unlikely to occur when the validity of alternative scores is investigated. In fact, previous research has revealed somewhat lower relations of speed with intelligence than that obtained with WMC [39], arguably an artifact of the narrow measurement model in that study that only comprised one type of reasoning task. Relations of speed with WMC and intelligence were highly comparable in other research using broader measurement models of intelligence incorporating a battery of sufficiently heterogeneous tasks [38]. The latter could be expected given that WMC constitutes a basis of intelligence [8,10,51]. Facing the time constraints in the current study, we decided to use the same WMC tasks as in previous research [39] that have been shown to possess highly satisfactory measurement models and validity for intelligence [52], and that were still brief enough to be included in this battery.

As a novel correlate, we additionally included two rapid serial visual presentation (RSVP) paradigms that have been used in the literature to estimate an attentional blink (AB) effect [53,54]. This effect could be of interest as it was theorized to reflect an attentional bottleneck at encoding into working memory. Given this is a processing-speed driven limitation in working memory, it could be predicted to be related with both, mental speed and working memory capacity. Moreover, it could be hypothesized that some of the mental speed scores are more strongly related with the AB effect than others. The latter would depend on the kind of—supposedly—speed-driven limitation that drives the AB effect. For instance, if general speed of processing had an effect, a pronounced relation with RT mean could be predicted. Conversely, if the AB effect was driven by occasional lapses of attention, slow RTs or errors would be most predictive.

**Figure 2:**
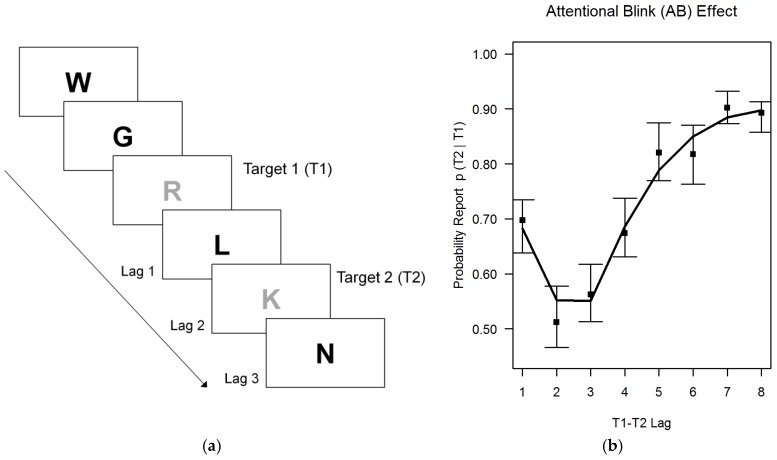
(**a**) Rapid Serial Visual Presentation (RSVP) Paradigm and (**b**) Attentional Blink (AB) effect.

In the RSVP paradigm, a stream of stimuli is presented in short succession (e.g., each 100 ms one stimulus), while participants are asked to report 2 target stimuli marked by a different font color (see Figure 2, left side). The probability of correctly reporting the second stimulus depends on the lag (target-to-target interval) between target 1 and target 2. Typically, participants can report both stimuli accurately when they are separated by a sufficiently long lag (e.g., 800 ms). However, when the second stimulus is presented shortly (e.g., 200–500 ms) after the first, recall probability is substantially reduced. This effect is known as the attentional blink (AB) effect. The average AB effect observed in this study is depicted on the right side of Figure 2 (median proportion of correctly recalled target 2 stimuli with 99% bootstrapped CI and a loess smoothed curve). Interestingly, previous research has revealed a high probability that participants can report the second stimulus, when it is presented directly (e.g., 100 ms) after the first stimulus. This effect is known as “lag-1 sparing” [55], and it speaks against an account in terms of perceptual impairments. Therefore, the AB effect has been classically interpreted as reflecting a capacity limitation when stimuli are encoded into working memory [56,57,58].

As can be seen in Figure 2, the AB effect observed in this study had a typical shape: After a lag-1 sparring (i.e., 100 ms after T1 presentation), a minimum of correctly recalled T2 stimuli is passed at lag 2 and 3 (i.e., 200–300 ms after T1 presentation). Then, the probability of correctly recalling T2 increases again, and reaches an asymptotic level again at lag 7 and 8 (i.e., 700–800 ms after T1 presentation). A number of alternative scores have been suggested to quantify the AB effect. Typically, only trials are used in which T1 could be correctly recalled, as it implies that T1 was processed. The commonly employed “AB magnitude” score denotes the difference between the minimum recall probability for T2 and the maximum recall probability for T2 [59], and has been shown to be a robust measure [60] that could be used to infer individual differences.

A couple of individual differences studies were conducted to test correlates of the AB effect with performance measures. Some research supports the notion that the AB effect reflects capacity limitations in working memory. For instance, in one study [59] WMC was inversely related with the magnitude of the AB effect. However, intelligence was only related with overall accuracy in that task. In another study [61], the probability of reporting the second target after correctly reporting the first target was correlated with WMC and intelligence. Yet another study [62] confirmed moderate relations of AB with WMC and processing speed. However, inverse relations of WMC with the AB effect were not supported in all studies [60]. The relationship of mental speed with WMC suggested in some studies [62] was not supported in other research, where the latter study actually comprised a set of different response time tasks [63]. Finally, inverse relations were reported for the AB effect with executive functions [61,64]. In summary, most studies addressing individual differences in AB magnitude investigated correlations with WMC or intelligence, supposedly because the task was classically conceptualized as a marker of capacity limitations. Additionally, executive functions were investigated as correlates. However, in spite of its obvious speediness only few studies addressed mental speed as a possible correlate. Another challenge arises from the fact that the AB magnitude is computed as a contrast score which tends to be unreliable in case of correlated constituents [65]. Consequently, some of the inconsistencies obtained in previous research may be due to insufficient reliability of the AB score. Please note that some studies used different variants of scoring the AB effect, which may have contributed to the apparent discrepancies of the observed relations.

### 1.4. Aims of This Study

The aim of this study was to replicate and extend previous research addressing the relation of mental speed with cognitive ability [30,38,39,40]. A particular focus was on alternative scoring methods and RT modeling approaches for mental speed tasks. As alternative performance metrics are differentially sensitive to different aspects of task performance, they could possess differential validity for cognitive ability. Specifically, the aims of this study were threefold:

1. **Investigate the validity of alternative speed scores.** A first aim was to test the validity of alternative scores and parameters computed from mental speed tasks that were suggested in the literature to predict cognitive ability. This included mean response times, within-person variability of response times, the proportion of slow responses, the validity of RT quantiles, and of commission errors. To judge the validity of the scoring method per se, a broad measurement model of mental speed was implemented that comprised indicators from all task types. Additionally to replicating relations with classical indicators of WMC, the AB effect was included as a correlate.
2. **Test moderation by task complexity.** We employed three classes of speed tasks that were supposed to differ in task complexity. Relations for each of the alternative speed scores were modeled separately within task types to test if validity was moderated by task type. Separate RT band analyses were computed for the task types to test if WPR effects were affected by task complexity.
3. **Evaluate process dissociation models for response times.** Parameterizations of the RT distributions with the ex-Gaussian and the diffusion model were fitted to test the validity of their parameters. This also aimed at addressing some of the discrepancies in parameter validity observed in recent studies.

## 2. Materials and Methods

### 2.1. Sample

The study was advertised in social media and by means of flyers. Inclusion criteria were an age between 18 and 40 years and sufficient knowledge of the German language. The sample comprised *N* = 129 young adults (*n* = 110 female), mostly university students (*n* = 121). On average, they were 22.0 years old (standard deviation (SD) = 3.1, range = 18–38). Research volunteers signed informed consent prior to participation, and received 15 Euros or partial course credit as compensation.

### 2.2. Materials and Procedure

**Mental speed tests.** In order to measure mental speed in a broad way, we used a battery of nine different clerical speed tests cf. [66], namely Search, Comparison, and Substitution tasks which were administered in a computerized fashion. Construction of the speed tests followed a matrix design where each type of task was combined with three different materials: numbers, letters, and symbols. The tasks were identical with those used in Schmitz and Wilhelm [39], however trials lists were longer in the present study, which is beneficial for a precise estimation of parameters in response time modeling [67,68]. Additionally, special response devices were used with customized mappings of the keys (e.g., for the Substitution task, see also below). Stimuli were presented on a 22′ TFT monitor. In all tasks, stimuli were presented in black ink on a light gray screen (RGB = 220, 220, 220). Error feedback was not provided.

In the Search tasks, stimuli were presented successively in the center of the screen and participants had to classify whether the currently presented stimulus was a target stimulus or not by pressing a right or left key, respectively. In the task with numbers, the target stimulus was a ‘3’, in the task with letters, it was an ‘A’, and in the task with symbols it was a smiley. In the task with letters and symbols, participants completed two blocks of 60 trials each (plus two warm-up trials which were not analyzed). In the task with numbers, they completed two blocks with a total of 135 trials (plus two warm-up trials for each block). Slightly more trials were presented for the sake of balancing trial lists and for some additional warm-ups as this was the first experimental task in the battery. Stimuli were presented in black LucidaSansRegular font (60 pt). After responding, the screen was cleared, and the next trial started after 500 ms.

In the Comparison tasks, each trial comprised two strings with three elements, which were presented horizontally aligned to the left and to the right of the center of the screen. Participants were asked to classify if both strings were identical or not by pressing a right or left key, respectively. In case of a difference, only one of the three depicted elements was exchanged. The task was administered with numbers, letters, and simple abstract symbols as stimuli. Individual stimuli were presented in black LucidaSansRegular font (50 pt). For each stimulus category, participants completed two blocks with 60 trials (plus two warm-up trials) each. After classification, stimuli were removed, and the next trial started after 500 ms.

In the Substitution task, a mapping list denoted how stimuli of one class (e.g., numbers) are mapped onto stimuli of another class (e.g., symbols). In the classical paper-and-pencil version of this task, participants were given lists with stimuli of the first class, and they were asked to write or draw the corresponding other stimulus next to the given stimulus. In the computerized version, the mapping table was presented on the lower part of the screen for the entire block, showing how nine stimuli of the first class were mapped onto nine stimuli of the second class. In each trial, one stimulus of the first class was presented on screen, and a button press was required on a special response device displaying the stimuli of the second class on the keys. All keys were in one row from left to right, and there was a larger “home” button in the middle closer to the participant. In each trial, participants were instructed to place the index finger of their dominant hand on the home button and while keeping it suppressed. After 500 ms, one stimulus of the first class appeared in the center of the screen and had to be classified by pressing the corresponding key with the index finger. After classification, the stimulus was removed from the screen (but the mapping table remained). If the participant did not press the home button again after another 500 ms, a reminder appeared. Participants had to complete three Substitution tasks, requiring the substitution of numbers with symbols, of letters with numbers, and of symbols with letters. Imperative stimuli were presented in black LucidaSansRegular font (50 pt). Special keyboards existed for all tasks and were exchanged in the pauses. Each task comprised two blocks of 60 trials (plus two warm-up trials) each.

The three task classes differ in task complexity. The simplest class of tasks is the Search task, as participants have to identify only one target stimulus. The Comparison task is of intermediate complexity, as it requires keeping in mind briefly three stimuli that have to be compared with the other set of stimuli. The substitution task is by far the most complex. In fact, the nine mappings can be expected to exceed the number of relations participants can keep reliably in working memory. Therefore, working memory can be assumed to be fully exploited, as participants will have to look-up mappings repeatedly.

**Working memory capacity paradigms.** Three recall-1-back tasks [52] were employed, with numbers, letters, and symbols as content following a matrix design. In the task variants with numbers and letters two to four rectangles were shown on screen horizontally aligned. Each run started with showing stimuli in all rectangles. Then, all the stimuli were removed, and one stimulus appeared unpredictably in one of the rectangles. When a stimulus was shown, participants were asked to type in the stimulus located prior to the current stimulus in the rectangle, and to remember the new stimulus that was currently shown. The task variant with symbols followed the same scheme. However, simple geometric figures were shown in the cells of a 3 × 3 grid. Participants had to identify by mouse click the position where a currently shown symbol had appeared the last time. Task requirements differed from run to run in terms of working memory loads (1, 2, 3, or 4 stimuli to remember) and updating (6, 9, or 12 updates).

All stimuli were shown for 3000 ms, which also corresponded with the response window for typing in the old stimulus and for memorizing the new stimulus (respectively, for indexing its position and remembering its new position in the task with symbols). Then, the stimulus was removed (but the rectangles or grid stayed on screen), and the next stimulus was presented after 500 ms. The task started with a training block that comprised three runs in which feedback was provided to make sure that participants understood the task logic. The following test block comprised 12 runs (with a total of 108 stimulus displays) without providing feedback. Partial credit scoring [69] was applied, i.e., the overall proportion of correct responses was determined.

**Rapid serial visual presentation tasks.** As it was not clear if AB scores would possess sufficient reliability for the modeling of individual differences, we employed two RSVP tasks. Thereby, parallel-test reliability could be estimated from the measurement model; and all relations could be estimated with a latent AB factor. Each RSVP trial comprised streams of 16 stimuli presented in short succession (with a lag of 100 ms). All stimuli were shown in the center of the screen in black font with the exception of the two target stimuli that were shown in red (RGB = 230, 0, 0). After completion of the stream, participants were asked to type in the first and the second target without time pressure. The first target stimulus was either presented as the third or as the fifth stimulus in the stream, the second target followed with an equal probability after one of 8 lags, i.e., from lag 1 (100 ms) to lag 8 (800 ms) after the first target stimulus. Each RSVP task comprised 3 blocks of 48 trials (plus one warm-up trial) each. Consequently, 144 trials were available for the analyses, 18 for each of the eight lags. As stimuli, one of the RSVP tasks comprised consonants, the other single digits. Stimuli were balanced to appear as targets and as distracters with comparable frequencies, direct stimulus repetitions were avoided. Stimuli were presented in LucidaSansRegular font (60 pt). Only trials in which the first target was correctly identified entered the analyses. The AB magnitude [59,60] was computed as the difference between the minimum and the maximum of the T2 report function (see Figure 2). In order to increase reliability of the difference score, lag 2 and 3 performance was collapsed for the minimum, and lag 7 and 8 were collapsed for the maximum.

**Procedure.** Data collection took place in form of group testing with up to six participants per testing session in a small-group testing room. Prior to administering the computerized tasks, a short paper-and-pencil test was administered in the first ten minutes (data will be reported elsewhere). Then, the experimental battery was started which comprised the above mentioned tasks in the following order: the RSVP task with letters; the Search tasks with numbers, letters, and symbols; the Comparison tasks with numbers, letter, and symbols; the Substitution tasks requiring substitutions of numbers with symbols, of symbols with letters, and of letters with numbers; the RSVP task with numbers; then a five minutes pause; the recall-1-back tasks with numbers, symbols, and letters, and finally some demographic questions. The entire study took about 1.5 h to complete.

### 2.3. Modeling Mental Speed

In line with the aim of this study, performance in the speed tasks was analyzed in alternative ways, as suggested in mental speed research and in the literature on RT modeling, e.g., [32], see also Introduction. Most of these scores are strongly correlated, as they are computed from the same response time data. However, some are differentially sensitive to specific characteristics of the RT distribution, which could affect validity for cognitive ability.

In the present paper, speed of responding was computed by averaging reciprocal response times. This metric corresponds with the way paper-and-pencil tests are scored, i.e., sum of correct responses in a given time. However, most experimental data derived from computerized tasks are analyzed by reporting the mean RT (*M*_RT_). As this metric can be biased by few extreme values, median RT (*Mdn*_RT_) or the mean of log-transformed RT (*M*_log(RT)_) were proposed as alternatives and also computed here. Further, we computed five equally-spaced quantiles of the RT distribution that offer the possibility to test differential validity of specific portions of the RT distribution (e.g., of slow responses, cf. WPR). Three scores tapping within-person variability were computed: the standard deviation (*SD*_RT_) the log-transformed variance (log(*V*_RT_)), and the interquartile range (IQR_RT_), where the latter is more robust in face of outliers than the former. Finally, the proportion of errors was computed and its probit transformation that normalizes the skewed error distribution. Additionally to these single score metrics of task performance, parameters of the ex-Gaussian parameterization and diffusion model were computed.

**Figure 3:**
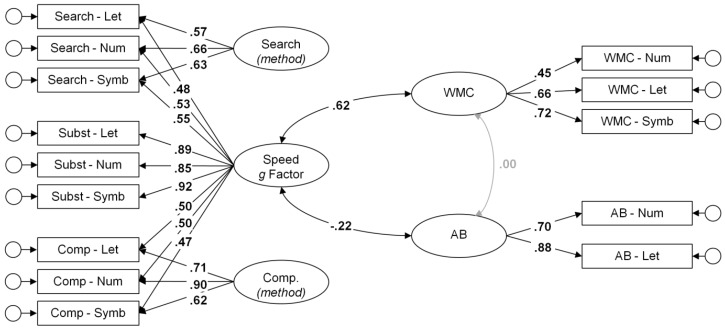
Latent Correlations of a Mental Speed General Factor with WMC and AB.

Validity of the performance scores was investigated by means of structural equation modeling. This allows investigating the conceptual validity of scores and the theoretically suggested processes captured by them, even when individual scores suffer from low reliability. To test one specific scoring algorithm (e.g., *M*_RT_), the score was computed for all speed tasks. A bifactor confirmatory factor analysis was fitted to the data (see Figure 3 for an illustration), with one broad *g* factor accounting for the communality across all speed tasks. Method factors were specified for the Search and for the Comparison tasks, whereas the Substitution tasks served as the reference method. This model was shown to offer the most parsimonious account of the structural relations of the employed speed tasks [39]. WMC and AB were specified as latent factors, too. The unstandardized loadings of the two AB indicators were constrained to equality. This constraint was made to facilitate convergence of the model when latent relations of the AB factor were low to virtually zero. However, in order to maintain a consistent family of models we kept the constraints in all analyses.

The correlation of the *g* factor of mental speed with WMC and AB was used to infer the validity of the scoring method employed to compute the observed indicators. A possible moderation by task complexity was tested analogously by computing latent factors for the three indicator tasks (Search, Comparison, and Substitution) of the same task type. Correlations of these task-specific speed scores were tested with WMC and AB. This allowed inspecting possible differences in the magnitude of the relations across task types.

Validity of the model-based parameters was tested following Schmiedek et al. [38]. Ex-Gaussian or diffusion model parameters were estimated for the speed tasks, and latent factors were specified for each type of parameter that captured the reliable portion of parameter variance. Residuals of parameters estimated from the same paradigm were allowed to be correlated, thereby reflecting parameter dependencies. In turn, the latent parameter factors could be used to infer the validity of the “true” parameter variances. Following Schmiedek et al. [38], relations of the model parameters with criterion variables were tested using latent regression and correlation analyses. Latent regression bares the advantage that unique relations can be determined for the correlated parameter factors (see Figure 5 for an illustration), however, at the potential risk of collinearity problems. Separate models were estimated for each task type to test differential validity of the parameters for different levels of task complexity cf. [39].

The data were analyzed with R [70], few missing data were imputed with Amelia [71]. The retimes package [72] was used for the ex-Gaussian analyses. To reduce the problems of computational dependencies, we chose the implemented two-step maximum likelihood procedure with bootstrapping that was recommended for small samples [73]. The function identifies *μ* and *σ* using a Gaussian kernel estimator in a first step and then *τ* is determined from bootstrapped values. Parameters of a simplified diffusion model were computed with EZ [74] which uses a closed-form expression to compute scores that correspond with drift rate, response criterion, and non-decision time of the diffusion model. This method was demonstrated to be suited for recovering individual differences even with moderate trial numbers [67,68]. The psych package [75] was used for psychometric analyses, and lavaan [76] for structural equation modeling. As some of the variables were not normally distributed, Satorra-Bentler robust maximum likelihood estimation was applied. Correlation matrices and data sets are provided in an online repository.^1^

## 3. Results

### 3.1. Preliminary Analyses

Two participants were excluded from the analyses, as data for some of the tasks were completely missing due to technical problems with the response devices, and data from one participant was removed due to an inconsistent response pattern. For the remaining participants, response time data were carefully checked for extreme values. Individual extreme value criteria were applied for persons and tasks, following the liberal Tukey [77] criterion. This means, responses were discarded if they were more extreme than three times the inter-quartile range on top of the 75 percentile, or three times the inter-quartile range below the 25 percentile, or below 200 ms. Additionally, responses from post-error trials were excluded, as they can be affected by post-error processes [78]. The proportion of excluded trials is displayed in Table 1.

Descriptive statistics of the speed tasks were highly comparable with those obtained in previous research with the tasks [39]. Apparently, the slightly longer trial lists did not substantially affect measurement characteristics. Generally, accuracy was very high, as would be expected in a speed task. Additionally, accuracy was comparable across all task types, irrespective of differences in task complexity. However, *M*_RT_ and *SD*_RT_ increased from the Search to the Comparison tasks and were largest in the Substitution tasks. Thereby, the predicted effects of task complexity on mean and variability were confirmed [20], cf. [45]. Performances in tasks of the same type but with different stimulus materials were comparable with respect to *M*_RT_ and *SD*_RT_ and the respective standard deviations across participants. If anything, tasks with symbols as stimuli tended to require longer processing times.

In line with predictions, all ex-Gaussian parameters yielded larger mean values and standard deviations across participants in more complex tasks. However, in each condition, the *μ* parameter was somewhat smaller than *M*_RT_, reflecting that the latter is affected by slow extreme values that are captured by the τ parameter in the ex-Gaussian distribution. In fact, both τ and σ yielded substantial values that increased with task complexity. A test of model fit is provided in Appendix A for the ex-Gaussian parameters, separately for all speed tasks. As we were primarily interested in individual differences, the correlation of model-implied and observed data points were checked for five quantiles of the RT distribution (i.e., 10%, 25%, 50%, 75%, and 90%). Almost perfect correlations indicated that model parameters could account for observed individual differences in task performance. Predictions concerning mean values were also confirmed for the diffusion model. Drift rates were reduced in the more complex Comparison task as contrasted with the simpler Search task. Additionally, there was an increase in response caution in the more complex Comparison task. Additionally, there was a noteworthy increase in non-decision time. The latter is remarkable, and could reflect higher encoding requirements in the Comparison tasks (see discussion). The diffusion model could not be fitted to the Substitution task, as this task has more than two response alternatives. As speed scores and parameters of the ex-Gaussian and diffusion model comprised a couple of extreme values (cf. Appendix A), the 2.5% most extreme values on each side were winsorized for the correlational analyses.

### 3.2. Validity of Alternative Speed Scores

Predictive validity of the alternative single scores of mental speed was tested using bifactor-type models with mental speed as a broad hierarchical factor across all speed tasks (see Figure 3). Details concerning measurement and fit of the structure equation models are provided in Table A1.

The correlates WMC and AB revealed highly satisfactory measurement models with high loadings and factor saturation (McDonald’s omega). The high loadings of the two RSVP indicators suggest that the AB magnitude was measured reliably in the present sample. Loadings and saturation were also highly satisfactory for the bifactor-measurement models of mental speed. Omega hierarchical was computed to infer the saturation of the general factor of mental speed.

Table 2 lists the latent relation of the general speed score with WMC and AB. It can be seen that most alternative speed scores revealed substantial relations with WMC in the range of 0.55 to 0.69. There was not much of a difference between the reciprocal speed score, the mean scores and the scores indexing variability. The correlation of the arithmetic mean was merely descriptively stronger than the ones obtained for the two robust scores of central tendency, i.e., the mean of log-transformed RTs and the median.

In line with predictions, there was a descriptive monotonous increase in validity from the fast 20% to the slow 80% quantile by 10 points, but the relation dropped again in the slowest 100% quantile (see Table 2). Generally, confidence intervals largely overlapped and the small differences observed were not statistically significant, neither for the different quantile scores nor for the other RT based scores. In contrast, error-based scores were not predictive of WMC at all, yielding relations close to zero.

Relations of the speed scores with the AB factor were also relatively homogeneous across all mean and variability scores, indicating that the AB effect is larger when responses were slower and more variable. However, the correlation in the magnitude of approximately *r* = 0.20 was just a third of that which was observed with WMC (and accordingly, the coefficient of determination was only a ninth). While AB magnitude revealed this moderate correlation with mental speed, its relation with WMC was estimated to be virtually zero (95% CI: −0.23; 0.24; in all SEM models). The relation with AB was hardly affected by the quantile of the RT distribution from which the speed score was computed. Finally, error-based speed scores revealed relations virtually around zero with AB magnitude.

### 3.3. Moderation by Task Complexity

A potential moderation of the speed-ability relation by task complexity was inspected by computing relations separately for the three types of speed tasks that were supposed to differ in complexity. To start with, RT-band analyses were computed for 20 increasing RT quantiles (5% steps, comprising 6–7 trials each). Closely following Larsen and Alderton [23], all trials were entered into the RT band analyses without exclusion of extreme values. Therefore, some extreme values might deteriorate validity in the slowest band. However, as the median RT was computed for each band, at least the two to three most extreme values were discarded. The fine-grained RT-band analyses were motivated by the rich information that they convey. Similarities and differences in the level and trajectories of different tasks’ validities can be graphically inspected. Additionally, overall level and slope can be used to foreshadow validities of some of the conventional scores and model parameters.

**Figure 4:**
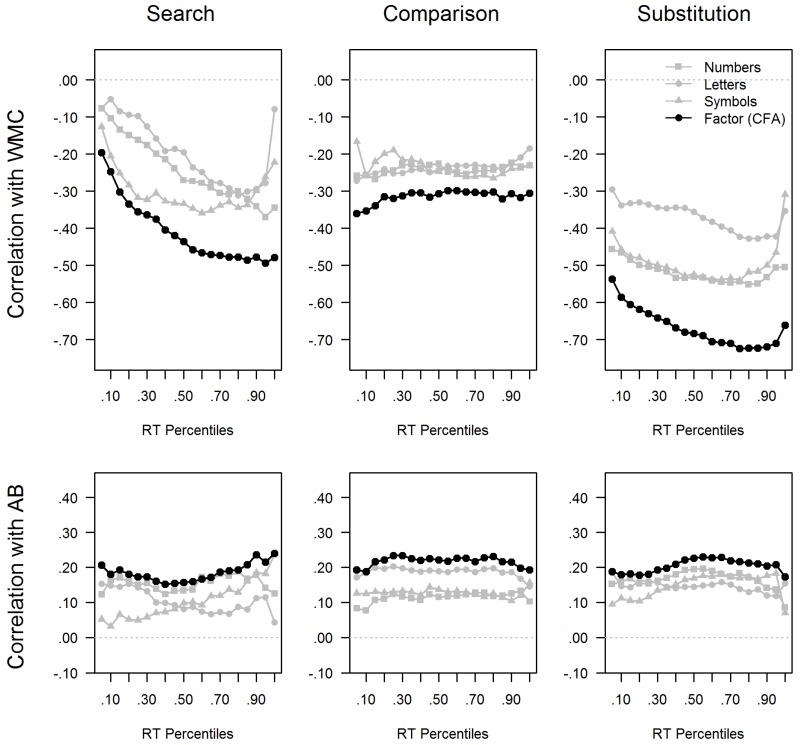
Correlations of RT Bands obtained in Mental Speed Tasks with WMC and AB. Factor (CFA) corresponds with a latent factor obtained in a confirmatory factor analysis using the three stimulus materials as observed indicators.

Figure 4 shows RT band analyses for each of the nine speed tasks (gray lines) when predicting WMC and AB. For the criterion variables, composite scores were computed from their standardized indicators. Thereby, the gray lines reflect observed correlations of the 20 RT bands of each speed task with the composite scores of WMC and AB. Additionally, analogous SEM analyses were conducted to dissociate the reliable task-specific components across RT bands from erratic effects that could occur in the indicators. A latent RT band factor was estimated across the three tasks of the same type for each RT band. Also WMC and AB were modeled as factors. The latent correlation of the RT-band factor with WMC and AB was estimated for each RT band, and is plotted in black for each speed task and criterion variable.

It is striking that RT-band trajectories were comparable within one type of speed task, whereas trajectories differed considerably across task types. In the Search tasks, there was a strong increase in predictive validity for WMC across RT bands from *r* = |0.20| to *r* = |0.50|, and this increase was observed in all Search tasks. There was also an increase in validity by approximately |20| points in the Substitution tasks, thereby a bit smaller than that observed in the Search tasks. However, in the Substitution tasks, all RT bands were generally more predictive, even the fastest RT bands (*r* = |0.50|). Again, comparable trajectories were observed for all Substitution tasks. Surprisingly, the Comparison tasks revealed only moderate validities (*r* = |0.30|), and these were not moderated by RT quantile. The absence of meaningful RT band moderation was observed in all three Comparison tasks. Differently from the previous findings, the RT band analyses for AB magnitude revealed a comparable pattern across all three types of speed tasks. In fact, the relation was only moderate (*r* = |0.20|) for all task types. Additionally, there was no evidence of any RT band moderation, and virtually all RT bands revealed comparable relations with AB magnitude.

In a next step, the speed scores previously tested in the bifactor models were now analyzed separately for each task. To this end, a latent factor was modeled across the three task of the same type, and latent correlations were determined with WMC and AB magnitude. Details of the measurement models and model fit statistics are given in Table A2. Relations with WMC and AB magnitude are listed in Table 3. Generally, RT based scores were correlated in a comparable magnitude within task. However tasks showed relations of different strength with WMC. The simple Search tasks were correlated around *r* = |0.40|, whereas the most complex Substitution tasks were correlated around *r =* |0.60| to |0.70|. However, contrary to predictions, Comparison tasks revealed the weakest relations around *r =* |0.30|. Relations of the variability scores were about comparable for the Search and for the Substitution tasks (in the range of *r =* |0.50| to |0.60|), but considerably smaller in the Comparison tasks (*r =* |0.30|).

In line with the RT band analyses, the Search tasks revealed a clear increase in relations with WMC across quantiles (of 19 points). A weaker increase was observed in the Substitution task from the 20% to the 80% quantile (of 9 points), and a drop again to the slowest quantile. In contrast to the latter, relations of the Comparison tasks were highly comparable across quantiles of the RT distribution. Validity of the error-based scores were around zero for all task types. Relations with AB-magnitude were generally moderate (*r =* |0.20|), but they varied less across task types. Interestingly, they were descriptively even stronger and consistent in the Comparison tasks than in the other two tasks. The AB-magnitude was not related with error-based scores in any of the tasks.

### 3.4. Process Dissociation Models for Response Times

**Figure 5:**
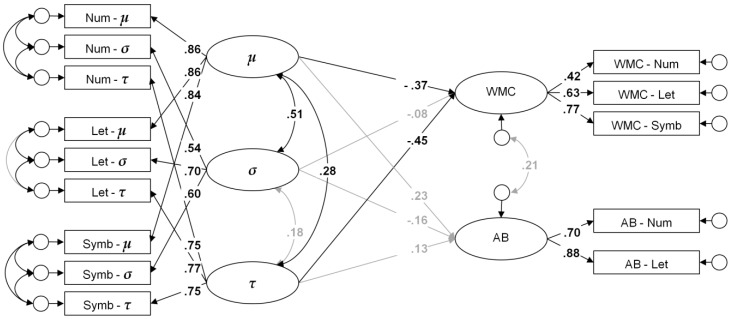
Parameters of the ex-Gaussian parameterization fitted to the Substitution tasks as predictors of WMC and AB. Significant parameters are displayed in black font.

The validity of parameters of the ex-Gaussian and of the diffusion model were tested concurrently using latent regression and correlation models (see Figure 5). Details concerning measurement model and model fit statistics are provided in Table A3.

Relations with WMC and AB magnitude are displayed in Table 4. Relations of the parameters of the ex-Gaussian and of the diffusion model were found to be somewhat lower than those for the single speed scores. Results obtained in regression and correlational analyses will be reported here in parallel, checking for the consistency of the estimates.

In the ex-Gaussian analyses, the τ parameter yielded the most consistent relations with WMC across all tasks and analyses. This implies that the skew of the RT distribution is negatively related with WMC. In the Search tasks, the τ parameter yielded significant relations with WMC and AB magnitude in regression and correlational analyses. Additionally, there was evidence in the correlational analyses that variable responding (σ) was related with low WMC. In the Comparison tasks, the mean response time (*μ*) revealed correlations with WMC and AB, whereas τ and σ were inconsistently correlated with low WMC and AB magnitude, respectively. Results of the regression analyses most likely reflected a collinearity effect due to the high correlation of the latent predictors *μ* and *σ*, and results should not be interpreted. In the Substitution tasks, regression and correlational analyses consistently indicated that mean (*μ*) and skew (τ) of the RT distribution are related with low WMC. Relations with AB magnitude were analogous, but more moderate, and missed inferential significance in most analyses.

In the diffusion model analyses, drift rate (ν) was the parameter that was most consistently related with WMC. However, additional parameters revealed significant effects, depending on task type. In the Search tasks, low response caution (*a*) was associated with high WMC, whereas in the Comparison tasks, low non-decision time (*T*_er_) was associated with WMC. Relations with AB magnitude were again more moderate, and missed significance in most of the cases.

## 4. Discussion

### 4.1. The Attentional Blink Effect

In this study, we employed rapid serial visual presentation (RSVP) paradigms to measure individual differences in the attentional blink (AB) effect. The design with two parallel tasks allowed estimating its reliability for the assessment of individual differences and investigating its relations with a set of mental speed measures and with WMC.

While contrast scores can be unreliable in many cases [65], the AB magnitude [59,60] scores computed independently from both RSVP paradigms loaded substantially on one common factor, thereby, giving evidence of its reliability. The AB magnitude factor was unrelated with WMC, whereas relations with a set of different RT based scores of mental speed were moderate. The absence of a relation of AB magnitude with WMC raises doubt that the capacity of WM is a limiting factor in the RSVP paradigm. Instead, (high) speed of processing seems to predict (small) attentional blink effects. Further, these relations were consistently observed across a battery of different speed tasks in the present study. This suggests that not task specificities but mental speed as the communality across tasks was responsible for the observed relations. However, the only moderate relation implies that mental speed can only offer a partial explanation of the AB magnitude.

The present results have to be evaluated in the light of the generally mixed evidence concerning AB correlates. For instance, some research supports the notion that the AB effect is independent of WMC [60] while other studies reported inverse relations [59,62]. In fact, an interesting dissociation has been reported in one of the latter studies [59], namely that AB magnitude is related with WMC but not with intelligence. Given the usually strong correlation of these constructs [3,51], it may be speculated that a speed confound present in many WMC tasks could have contributed to the observed relation with AB magnitude. In fact, relations of AB with mental speed have been confirmed in some research [62], although evidence in this direction has been disconfirmed in other work [63]. This negative result is surprising given that a broad battery of speed tasks was administered in that study similar to the ones employed in the current study.

Another interesting finding in the present study was the absence of a quantile moderation of the speed-AB relation. It was striking that all RTs predicted AB magnitude in the same moderate magnitude across RT quantiles, and this was consistently found for all speed tasks. Quantile moderation was even absent for speed tasks in which clear quantile moderation was observed for the speed-WMC relationship. The absence of task moderation suggests that the speed-AB relation is indeed driven by a common speed factor and not specific requirements of the speed tasks. Additionally, the absence of quantile moderation implies that the general level or position of the RT distribution (corresponding with the ex-gaussian *μ* parameter) is more relevant than its skew or proportion of extreme values (corresponding with the *τ* parameter). The latter rules out that lapses, of attention are responsible for individual differences in the attentional blink effect, whereas an account in terms of general speed of processing is supported. Thereby, the absence of quantile moderation is another characteristic that distinguishes the AB effect from WMC and intelligence, as the latter frequently show quantile moderation, at least when the speed tasks are more complex cf. [24,28,29].

### 4.2. Validity of Alternative Speed Scores

Bifactor models were fitted to all single scores of mental speed investigated in this study. Highly satisfactory fit indices and substantial loadings support the notion of mental speed as a hierarchical construct. Thereby, it is confirmed that the investigated speed tasks reflect a common factor of mental speed as well as task-specific requirements shared only by the tasks of the same type. In fact, comparable results have been obtained for conventional speed scores (1/RT) using paper and pencil tests and for mean response times (e.g., *M*_RT_) using computerized tasks [3,4,5]. The present results complement this evidence by showing that a hierarchical structure holds as well for a number of alternative performance metrics, such as variability indices, and even for error-based scores. Comparable factor saturation across quantiles of the RT distribution additionally suggests that there is no substantial (de-)differentiation across the RT distribution.

In line with previous research [39], the RT-based single scores of mental speed revealed relations with WMC in comparable magnitude. Similarly, relations with AB magnitude were highly comparable across tasks, although they were generally weaker than those obtained with WMC. RT mean scores based on natural RTs (*M*_RT_) and their robust counterparts (*Mdn*_RT_; *M*_log(RT)_) revealed almost identical correlations with WMC and AB magnitude. This implies that the validity is not substantially affected by slow extreme values, contradicting postulates in some earlier research [21,22].

Variability scores were also related with both criterion variables. It is of note that the standard deviation and log-transformed variance scores yielded comparable fit and predictive validity. In fact, a linear relation of the standard deviation would imply a nonlinear relation of the log-transformed variance, and vice versa. It is unresolved whether this reflects a measurement or a modeling issue. Clearly, this is unsatisfactory from a theoretical perspective. However, from a pragmatic perspective, it suggests that both scoring alternatives function comparably well in terms of predictive validity.

The finding that IQR was more predictive than the standard deviation of RT (*SD*_RT_) showed that validity of the variability metrics is not driven by slow extreme values which could only affect *SD*_RT_ but not IQR. However, variability indices revealed descriptively weaker relations with criterion variables than mean RT scores. Thereby, a superiority of variability indices over mean scores postulated in some earlier research [22,40] was not supported. The present results are in line with a recent meta-analysis conducted across studies using different Hick tasks, which showed that variability scores were not superior over mean response times [14].

In contrast to the generally predictive RT-based scores, error-based scores did not reveal relations with WMC or AB magnitude at all, as can be inferred from their random fluctuation around zero. It is of note that the measurement models of the error score were satisfactory in terms of model fit and loadings. Consequently, insufficient reliability cannot be the reason for the absence of relations. The probit transformation that normalizes the heavily skewed error distributions did not ameliorate the problem either. It can be speculated that individual differences in error rates reflected other factors than impaired ability, e.g., individual differences in response caution or some sort of distractibility that is unrelated with the WMC and AB criterion variables.

### 4.3. Moderation by Task Complexity

We used three class types that were assumed to differ in complexity. In fact, preliminary analyses supported that Search tasks were the simplest, Comparison tasks were of intermediate complexity, and Substitution tasks were the most complex in terms of task performance. Mean and standard deviation of the RTs increased across task types, whereas error scores were about comparable. The predicted validity moderation by task complexity was investigated by inspecting relations with WMC and AB within task types.

RT band analyses revealed differences between task types in terms of overall level of validity and in terms of a quantile moderation of the relations with WMC. A strong worst performance rule (WPR) effect [23,24] occurred in Search tasks and to a lesser extent in Substitution tasks. However, the latter were generally much more predictive, even the fastest response quantiles. Consequently, the slowest RTs in Substitution tasks were the most predictive of all, even though the increase across quantiles was not as large as in the Search tasks. In contrast, the Comparison tasks revealed the weakest relation with WMC, and there was no evidence of a quantile moderation. The trajectories of the three tasks of the same type were highly comparable, supporting that the observed differences between task types are indeed dependent on task type. However, differently from previous findings [28,29], validity and WPR effects were not monotonously related with task complexity: First, the supposedly simplest Search task revealed the most pronounced quantile moderation, although WMC requirements or a high loading on a *g* factor of intelligence are unlikely for this task type. Second, the Comparison tasks revealed the weakest relations and no quantile moderation, although they were shown to be of intermediate task complexity in terms of mean response times.

The latent relations for the speed scores were accordingly revealing moderate relations with WMC for Search and strong correlations with Substitution, but the lowest relations with Comparison tasks. Correlations across alternative RT mean and variability scores within task class were highly comparable, which means that all investigated RT scores revealed comparable differences in validity across task types. In contrast, error-based scores were not predictive of WMC or AB magnitude in any of the three task types.

Both tasks that showed a quantile moderation also revealed higher validity of the RT variability scores. This implies that RT variation within person was related with the predicted construct. Of course, RT variation was observed also in the Comparison tasks (see Table 1) but this variation seemed to be driven by other factors. In turn, this rules likely that observed task difficulty is not a unitary factor but differently composed across types of speed tasks. However, given that all tasks load on a general factor of mental speed, the notion of mental speed as a hierarchical construct [3,4,5] is supported. In turn, this implies that task specificity can moderate the relation with WMC (and other constructs) to the degree it comprises a relevant requirement.

### 4.4. Process Dissociation Models for Response Times

The latent parameter factors of the ex-Gaussian and the diffusion model revealed a differential pattern of loadings. Generally, the ex-Gaussian *τ* and *μ* parameters and the diffusion model drift rate revealed the most consistent relations across task types and analyses. Correlations of these parameter factors with WMC were about in the same magnitude as those observed with conventional speed factors (cf. Table 3 and Table 4). Somewhat smaller correlations were observed in the Substitution tasks where the relation of speed with WMC was distributed across two parameters (*μ* and *τ*). However, relations were not confined to the above-mentioned parameters, and some of the relations were not significant, depending on type of analysis.

In the ex-Gaussian analyses, the *τ* parameter revealed correlations with WMC in all task types. However, there was some evidence that the σ parameter is also correlated with WMC in the simple Search task, thereby confirming previous research [40] conducted with comparably simple speed tasks, namely, numerosity and odd-even judgments of presented numbers. The *τ* parameter was also related with WMC in the complex Substitution task, however, there was no increase in validity with complexity, as observed in a study with tasks that require different levels of working memory [30]. In contrast, when complexity was higher in this study, *μ* gained validity additionally to *τ*. This confirmed predictions derived from continuous sampling models [45,46] (see next paragraph) and replicated some previous findings [39], while reversed effects were reported in other studies [30].

In the diffusion model analyses, drift rate (ν) was the parameter that was most consistently related with WMC across task types and analyses. In fact, the model offers a parsimonious account of the previously described effects in the ex-Gaussian parameters [45,46]. In simple tasks, drift rates of all persons would be relatively steep and with small variance. Consequently, individual differences would be reliably reflected only in the tail of the distribution. With increasing task difficulty, drift rates would be flatter and there would be considerably more variance between persons. As a consequence, the position of the RT distributions (*μ*) would gain reliability additionally to its skew (*τ*).

However, relations with WMC observed in this study were not confined to drift rates. Criterion setting (*a*) was inversely related with WMC in the simple Search task. In fact, a small inverse relation of this parameter has been observed in previous research [38]. This might reflect that highly able persons commit fewer errors and consequently respond less cautiously. Or, it might reflect that able persons realize that tasks are simple, and adjust their response criterion accordingly. In contrast, in the more complex Comparison tasks, there was no relation with response caution. However, non-decision time (*T*_er_) was predictive of WMC, additionally to drift rate. Non-decision time is conceived as reflecting processes outside the actual decision phase [41], such as encoding of stimuli or motor execution. Given that response requirements were highly comparable with that in the Search task (i.e., to press one of two buttons), the non-decision time effects likely reflect individual differences in stimulus encoding, which was a particular requirement in the Comparison tasks. In turn, while speed of encoding (*T*_er_) could be dissociated from the efficacy of decision making (ν), both processes may be related with WMC. However, the finding that non-decision time was related with WMC in a comparable magnitude as drift rates should be handled with caution, given that these effects may be highly task specific, and given that virtually all previous studies have identified drift rate as the parameter which is most consistently related with cognitive ability [38,39,40].

As some of the results for the ex-Gaussian and the diffusion model obtained in this study were not as consistent as in previous research, we additionally tested multiple regression models of the three observed moments that are used to compute diffusion model parameters in EZ [74], namely RT mean and variance and error rate. This allowed testing if the major results could be replicated without applying potentially error-prone models to individual RT distributions. Analogous SEM models were fitted as for the ex-Gaussian and diffusion model parameters. Latent factors were specified for RT mean (*M*_RT_) and variance (*V*_RT_), and for error rate (*Error Rate*). Model fit and relations with WMC and the AB magnitude are provided in Appendix Table A3. *M*_RT_ and *V*_RT_ were positively correlated across persons in all task types, conceptually replicating the results for the ex-Gaussian *μ* and *σ* parameters. This means persons who responded slowly also respond variably. Additionally, *M*_RT_ and *Error Rate* were negatively related, indicating a speed-accuracy trade-off. Persons who responded more slowly than others committed less errors, and vice versa. With respect to validity, *V*_RT_ predicted WMC in the simple tasks, whereas *M*_RT_ gained validity in more complex tasks, conceptually replicating findings for the ex-Gaussian parameters. However, *V*_RT_ as a global measure of variability does not distinguish between different sources of variability, such as *σ* and *τ* in the ex-Gaussian parameterization. Further, *M*_RT_ subsumes separable processes that are distinguished in the diffusion model, such as drift rate, response caution, and non-decision time. In summary, the analyses of the moments of task performance were apparently more robust and results more consistent across task types. Important findings from the analyses of ex-Gaussian and diffusion model parameters were conceptually replicated. However, the moments do not distinguish between theoretically meaningful sources of individual differences.

### 4.5. Limitations and Future Research

This study has a number of limitations that call for caution when interpreting the findings. To start with, the sample size was only moderate and comprised mostly students. Hence, generalizability should be tested with more diverse samples. Additionally, more heterogeneous samples would reduce the risk of range restrictions in the investigated ability constructs. Three types of speed tasks were employed in this study which revealed characteristics speed-WMC relations in terms of general level and trajectory across quantiles. Given that the relations with criterion variables depended on task type, a replication with yet different tasks would be desirable. The pattern obtained for the Comparison tasks did not meet predictions. In spite of its intermediate task difficulty it was only moderately related with WMC and there was no quantile moderation in the present sample. However, these findings should be interpreted with caution, as the same task was much better in line with predictions of previous research [39].

Additionally, it needs to be discussed that observed task difficulty was used here as a proxy of complexity. The latter is a theoretical concept that builds on the number and requirements of assumed mental operations. Naturally, one could think of instances when observed difficulty and actual task complexity do not correspond. Whereas task difficulty was observed as predicted (cf. Table 1), the differential patterns of relations warns us not to use the terms “difficulty” and “complexity” interchangeably. This leads to another limitation in the present study, namely that effects of task complexity were investigated by comparing different tasks that varied in observed difficulty. However, the differential pattern of relations observed for the respective tasks suggests that tasks differed in a number of requirements, which complicates the comparison across task types. Therefore, complexity should be experimentally manipulated within task in future research in order to increase internal validity.

Additionally, it has to be discussed that speed tasks were presented in the order of increasing complexity in this study. Although all tasks were relatively short and re-starting the next task after each pause was self-paced, it cannot be ruled out that slower responding in the more complex task might as well reflect fatigue or decreasing motivation. Therefore, a confounding of complexity and serial position should be avoided in future research.

Overall, the speed–WMC correlations were strong in this study. In part, this may result from a speediness of many WMC paradigms cf. [79]. Removing or at least reducing time pressure from the WMC tasks would help arrive at less biased estimates. In this study, we assessed cognitive ability only with a set of WMC tasks, as working memory capacity and intelligence have been shown to be substantially related in previous research [2,8,9,10]. Future research should test relations as well with alternative WMC tasks and conventional, broad intelligence tests. The results obtained for the attentional blink effect suggest that it is independent from binding-updating measures of WMC. Additionally, it is only moderately related with various aspects of mental speed performance, in spite of its highly reliable measurement. More research is required to understand what exactly is reflected in the AB effect from an individual differences perspective. Additionally, a number of alternative scoring methods were suggested for the paradigm, but their psychometric characteristics have to be evaluated.

This study aimed at testing alternative scoring methods for mental speed tasks. It turned out that most RT based scores revealed comparable relations with WMC and AB. However, given the moderate sample size, this study lacked statistical power to test the significances of the mostly small differences of the highly related RT scores. Post-hoc power tests were performed using the simsem R package [80]. The bifactor measurement model of the speed score (1/RT) depicted in Figure 3 was used as the parent model, and nested models were specified in which the speed-WMC relations were numerically fixed at decreasing values. The power to detect a significant difference in the latent correlation of 5, 10, 20, and 30 points were 0.07, 0.23, 0.53, and 0.93, respectively. Obviously, the power was low to confirm the mostly small correlational differences observed in this study. Therefore, most results were reported in a descriptive way. However, some of the core findings have been demonstrated in other studies e.g., [30,38,39,40], and convergence of independently sampled data is the most convincing piece of evidence. Additionally, rather than pointing to small differences with arguably little practical relevance, the take-home message from this study should be that the validity of most conventionally used RT-based speed scores was about comparable.

The relations for the latent parameters appeared to be less specific in the current sample than in previous research [38,39]. In part, this may result from the moderate sample size in this study relative to the complexity of the model. Additionally, the relations obtained for some parameters differed in magnitude between regression and correlational analyses. This can be expected given the overlap of parameters that would be controlled for in the regression analyses. However, when the direction is reversed (as in the ex-Gaussian model of the Comparison task), the results appear suspicious. Again, a larger sample with a more stable covariance matrix would be desirable, and the robustness of parameter relations across independent studies need to be checked. Further, a larger number of indicator tasks as in [38] could be beneficial for the measurement models.

### 4.6. Summary and Conclusions

Mental speed can be adequately described as a hierarchical construct. This does not only hold on for conventional mean RT scores, but as well for RT variability, for errors, and for a range of different RT quantiles. Task performance in mental speed tasks is moderately to highly related with WMC and to a considerably lesser extent with AB magnitude, whereas WMC and AB magnitude were virtually zero correlated. Most RT-based mean and variability scores revealed comparable relations with the criterion variables, whereas error-based scores did not reveal validity for the criterion scores at all.

RT band analyses revealed different patterns for speed-WMC relation for each task type, whereas tasks of the same type revealed comparable patterns. This suggests task specific effects and supports the hierarchical structure of the speed construct. In contrast, there was no quantile moderation of the speed-AB relation in either of the tasks. Relations of the investigated scoring methods revealed comparable relations with the WMC and AB constructs within task class, but differed between task classes.

Process dissociation models for response times with latent parameter factors revealed differential patterns of relations for the parameters, in part depending on task type and analysis methods. In the ex-Gaussian analyses, *τ* was the parameter that revealed the most consistent inverse relations with WMC across tasks and analysis methods. There was some evidence that *σ* is related with WMC in simple tasks, whereas *μ* gained validity with increasing task difficulty. In the diffusion model analyses, the drift rate yielded the most consistent effects across tasks and analyses. The response criterion (*a*) was inversely related with WMC in simple Search tasks, while non-decision time (*T*_er_) was negatively related with WMC in the Comparison tasks.

## Figures and Tables

**Table 1:** Descriptive statistics of response time (RT) scores and model parameters.

Task/Material	Excluded Trials		Descriptive Statistics			Ex-Gaussian Model			Diffusion Model		
	Post-Err	Extreme	*M*_RT_	*SD*_RT_	Acc	*μ*	*σ*	*τ*	*a*	*ν*	*T*_er_
***Search***											
Numbers	0.035 (0.041)	0.009 (0.030)	373 (32)	74 (14)	0.971 (0.023)	307 (33)	34 (11)	66 (19)	2.95 (0.54)	0.013 (0.002)	263 (23)
Letters	0.068 (0.080)	0.027 (0.059)	372 (33)	78 (17)	0.954 (0.041)	300 (28)	32 (12)	71 (20)	2.87 (0.49)	0.012 (0.002)	257 (21)
Symbols	0.063 (0.032)	0.011 (0.013)	459 (39)	78 (17)	0.945 (0.031)	388 (33)	54 (17)	66 (19)	2.86 (0.35)	0.010 (0.001)	332 (21)
***Comparison***											
Numbers	0.053 (0.034)	0.002 (0.006)	838 (111)	204 (63)	0.949 (0.033)	655 (71)	80 (29)	185 (60)	4.40 (0.89)	0.007 (0.001)	549 (57)
Letters	0.074 (0.041)	0.005 (0.008)	967 (148)	234 (65)	0.930 (0.041)	751 (96)	91 (29)	212 (67)	4.50 (0.91)	0.006 (0.001)	645 (79)
Symbols	0.100 (0.054)	0.020 (0.017)	1143 (156)	303 (74)	0.917 (0.057)	849 (95)	138 (43)	286 (88)	5.01 (0.95)	0.005 (0.001)	726 (72)
***Substitution***											
Num → Sym	0.041 (0.032)	0.015 (0.019)	1343 (192)	369 (92)	0.973 (0.025)	1002 (175)	154 (62)	349 (107)	-	-	-
Let → Num	0.036 (0.030)	0.007 (0.009)	1243 (190)	332 (87)	0.970 (0.031)	947 (167)	124 (54)	311 (105)	-	-	-
Sym → Let	0.005 (0.009)	0.036 (0.030)	1158 (126)	300 (75)	0.967 (0.029)	887 (119)	111 (42)	275 (79)	-	-	-

All statistics given are M (SD) across persons. Post-Err = post-error trials. Extreme = extreme values according to liberal Tukey (1977) criterion. *M*_RT_ = mean response time, *SD*_RT_ = within-person standard deviation in RT, Acc = Accuracy.

**Table 2:** Correlations of latent mental speed general factor with working memory capacity (WMC) and AB.

Score	WMC	AB
***Speed***		
1/RT	**0.62 [0.46;0.78]**	**−0.22 [−0.39;−0.04]**
***Response Time***		
*M*_RT_	**−0.67 [−0.84;−0.51]**	**0.22 [0.04;0.40]**
*M*_log(RT)_	**−0.65 [−0.81;−0.49]**	**0.22 [0.04;0.40]**
*Mdn*_RT_	**−0.66 [−0.83;−0.49]**	**0.23 [0.04;0.41]**
***RT Quartiles***		
Q_0.20_	**−0.59 [−0.77;−0.41]**	**0.20 [0.01;0.38]**
Q_0.40_	**−0.63 [−0.80;−0.46]**	**0.22 [0.03;0.40]**
Q_0.60_	**−0.68 [−0.85;−0.51]**	**0.23 [0.04;0.41]**
Q_0.80_	**−0.69 [−0.86;−0.53]**	**0.22 [0.03;0.41]**
Q_1.00_	**−0.62 [−0.77;−0.47]**	**0.20 [0.00;0.39]**
***RT Variability***		
*SD*_RT_	**−0.58 [−0.74;−0.42]**	0.15 [−0.06;0.35]
log(*V*_RT_)	**−0.55 [−0.71;−0.39]**	0.18 [−0.02;0.38]
IQR	**−0.67 [−0.84;−0.51]**	0.18 [−0.03;0.39]
***Errors***		
Error Rate	0.07 [−0.17;0.31]	0.04 [−0.14;0.21]
Probit (Error)	0.10 [−0.19;0.38]	0.02 [−0.17;0.20]

Significant relations are printed in boldface. Boundaries of the 95% confidence intervals are given in square brackets.

**Table 3:** Correlations of latent mental speed task-specific factors with WMC and AB.

Score	Search		Comparison		Substitution	
	WMC	AB	WMC	AB	WMC	AB
***Speed***						
1/RT	**0.41 [0.21;0.60]**	−0.16 [−0.35;0.03]	**0.29 [0.09;0.49]**	**−0.22 [−0.42;−0.03]**	**0.63 [0.48;0.78]**	**−0.21 [−0.39;−0.03]**
***Response Time***						
*M*_RT_	**−0.44 [−0.63;−0.25]**	**0.19 [0.00;0.37]**	**−0.31 [−0.53;−0.09]**	**0.22 [0.03;0.41]**	**−0.68 [−0.84;−0.52]**	**0.21 [0.02;0.40]**
*M*_log(RT)_	**−0.43 [−0.62;−0.23]**	0.17 [−0.02;0.36]	**−0.30 [−0.51;−0.09]**	**0.22 [0.03;0.41]**	**−0.66 [−0.81;−0.50]**	**0.21 [0.03;0.40]**
*Mdn*_RT_	**−0.43 [−0.63;−0.24]**	0.17 [−0.02;0.36]	**−0.32 [−0.54;−0.10]**	**0.22 [0.03;0.41]**	**−0.67 [−0.83;−0.50]**	**0.22 [0.03;0.41]**
***RT Quartiles***						
Q_0.20_	**−0.33 [−0.56;−0.10]**	0.15 [−0.05;0.34]	**−0.33 [−0.56;−0.11]**	**0.22 [0.03;0.42]**	**−0.60 [−0.78;−0.42]**	**0.19 [0.00;0.38]**
Q_0.40_	**−0.40 [−0.60;−0.20]**	0.13 [−0.06;0.33]	**−0.33 [−0.55;−0.10]**	**0.23 [0.03;0.42]**	**−0.65 [−0.81;−0.48]**	**0.21 [0.03;0.40]**
Q_0.60_	**−0.48 [−0.67;−0.29]**	0.17 [−0.03;0.36]	**−0.31 [−0.54;−0.09]**	**0.21 [0.02;0.41]**	**−0.69 [−0.85;−0.53]**	**0.22 [0.03;0.41]**
Q_0.80_	**−0.48 [−0.66;−0.31]**	**0.19 [0.01;0.38]**	**−0.30 [−0.53;−0.08]**	**0.23 [0.04;0.42]**	**−0.70 [−0.86;−0.54]**	**0.21 [0.01;0.40]**
Q_1.00_	**−0.52 [−0.71;−0.33]**	**0.22 [0.02;0.43]**	**−0.23 [−0.44;−0.02]**	0.19 [−0.02;0.39]	**−0.63 [−0.78;−0.48]**	0.18 [−0.02;0.37]
***RT Variability***						
*SD*_RT_	**−0.55 [−0.72;−0.37]**	0.18 [−0.01;0.37]	**−0.28 [−0.49;−0.06]**	0.19 [−0.01;0.39]	**−0.57 [−0.73;−0.41]**	0.13 [−0.07;0.34]
log(*V*_RT_)	**−0.53 [−0.69;−0.37]**	0.17 [−0.03;0.36]	**−0.25 [−0.45;−0.05]**	0.20 [−0.01;0.40]	**−0.54 [−0.70;−0.38]**	0.17 [−0.04;0.37]
IQR	**−0.54 [−0.69;−0.39]**	0.17 [−0.04;0.38]	**−0.31 [−0.53;−0.08]**	0.18 [−0.02;0.39]	**−0.65 [−0.81;−0.48]**	0.17 [−0.05;0.39]
***Errors***						
Error Rate	0.02 [−0.22;0.27]	0.05 [−0.18;0.28]	−0.09 [−0.33;0.14]	−0.03 [−0.23;0.17]	0.11 [−0.12;0.35]	0.03 [−0.15;0.21]
Probit (Error)	−0.01 [−0.25;0.24]	0.08 [−0.17;0.33]	−0.09 [−0.34;0.16]	−0.03 [−0.24;0.17]	0.16 [−0.13;0.44]	0.00 [−0.18;0.18]

Significant relations are printed in boldface. Boundaries of the 95% confidence intervals are given in square brackets.

**Table 4:** Latent variables regression and correlation models with WMC and AB.

Task/Parameter	Correlations of Predictors		Regression Weights		Correlations	
			WMC	AB	WMC	AB
***Ex-Gaussian Model***						
***Search***	***μ***	***σ***				
***μ***	-	-	−0.01 [−0.31;0.29]	0.31 [−0.02;0.64]	−0.19 [−0.43;0.04]	0.04 [−0.16;0.24]
***σ***	**0.67 [0.48;0.85]**	-	−0.25 [−0.56;0.07]	**−0.42 [−0.80;−0.04]**	**−0.34 [−0.53;−0.15]**	−0.15 [−0.41;0.10]
***τ***	0.04 [−0.18;0.26]	0.18 [−0.11;0.47]	**−0.49 [−0.68;−0.30]**	**0.36 [0.14;0.58]**	**−0.54 [−0.72;−0.35]**	**0.30 [0.09;0.51]**
***Comparison***	***μ***	***σ***				
***μ***	-	-	**−0.55 [−0.95;−0.15]**	0.10 [−0.30;0.51]	**−0.32 [−0.56;−0.08]**	**0.25 [0.05;0.44]**
***σ***	**0.83 [0.75;0.91]**	-	**0.50 [0.02;0.99]**	0.17 [−0.31;0.64]	−0.14 [−0.38;0.10]	**0.26 [0.05;0.46]**
***τ***	**0.70 [0.59;0.80]**	**0.68 [0.51;0.85]**	−0.26 [−0.56;0.03]	0.01 [−0.37;0.38]	**−0.31 [−0.51;−0.11]**	0.19 [−0.01;0.40]
***Substitution***	***μ***	***σ***				
***μ***	-	-	**−0.37 [−0.58;−0.15]**	0.23 [−0.01;0.47]	**−0.53 [−0.73;−0.33]**	0.19 [−0.00;0.38]
***σ***	**0.51 [0.35;0.67]**	-	−0.08 [−0.32;0.16]	−0.16 [−0.44;0.12]	**−0.35 [−0.60;−0.09]**	−0.02 [−0.25;0.22]
***τ***	**0.28 [0.04;0.52]**	0.18 [−0.10;0.47]	**−0.45 [−0.65;−0.24]**	0.13 [−0.10;0.36]	**−0.56 [−0.74;−0.39]**	0.16 [−0.05;0.38]
***Diffusion Model***						
***Search***	***a***	***ν***				
***a***	-	-	**−0.31 [−0.53;−0.09]**	0.01 [−0.21;0.23]	**−0.36 [−0.58;−0.14]**	0.06 [−0.16;0.27]
***ν***	−0.02 [−0.23;0.19]	-	**0.38 [0.20;0.57]**	−0.20 [−0.43;0.03]	**0.34 [0.17;0.52]**	−0.15 [−0.36;0.06]
***T_er_***	**0.23 [0.03;0.44]**	**0.29 [0.10;0.48]**	−0.16 [−0.38;0.06]	0.18 [−0.07;0.42]	−0.12 [−0.35;0.11]	0.12 [−0.07;0.32]
***Comparison***	***a***	***ν***				
***a***	-	-	0.03 [−0.20;0.26]	0.02 [−0.22;0.26]	−0.18 [−0.40;0.05]	0.15 [−0.05;0.36]
***ν***	**−0.27 [−0.45;−0.09]**	-	**0.24 [0.02;0.46]**	−0.13 [−0.34;0.09]	**0.32 [0.12;0.51]**	−0.19 [−0.39;0.00]
***T_er_***	**0.53 [0.39;0.68]**	**−0.34 [−0.49;−0.19]**	**−0.26 [−0.49;−0.04]**	0.18 [−0.06;0.43]	**−0.33 [−0.53;−0.13]**	**0.24 [0.05;0.43]**

Significant relations are printed in boldface. Boundaries of the 95% confidence intervals are given in square brackets.

**Figure A1:**
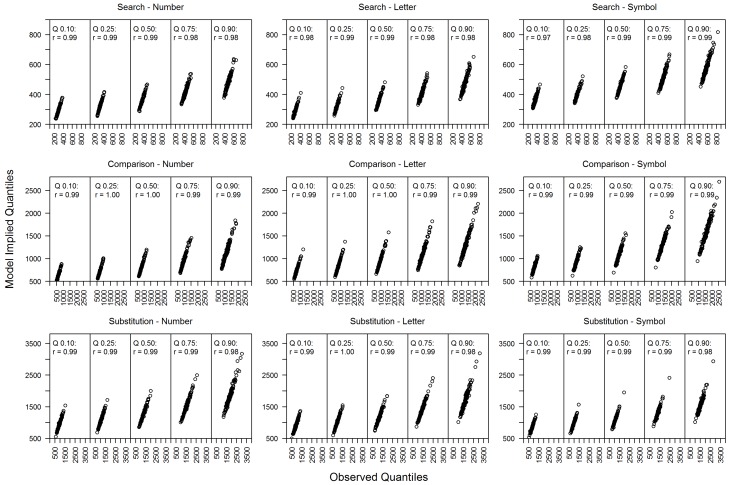
Scatterplots and correlations across participants of model implied and observed RTs for five quantiles (0.10, 0.25, 0.50, 0.75, and 0.90) of the RT distribution.

## Footnotes

1 https://osf.io/4avtd/?view_only=6aead5c066ff474ca5bd4860e0051ac2.

## Appendix B

**Table A1:** Measurement models and model fits of mental speed general-factor models.

Model	Measurement Model		Model Fit				
	ω	Mean Loadings (Range)	χ^2^ (*df)*	*p*	RMSEA [CI]	SRMR	CFI
***Only Correlates***							
WMC	0.43	0.61 (0.44;0.75)	8.07 (5)	0.15	0.07 [0.00;0.15]	0.03	0.97
AB	0.39	0.61 (0.44;0.75)	-	-	-	-	-
***+Speed***							
1/RT	0.69	0.63 (0.47;0.92)	117.41 (69)	0.00	0.07 [0.05;0.10]	0.08	0.95
***+Response Time***							
*M*_RT_	0.73	0.65 (0.49;0.92)	103.86 (69)	0.00	0.06 [0.04;0.09]	0.07	0.97
*M*_log(RT)_	0.71	0.64 (0.48;0.92)	110.91 (69)	0.00	0.07 [0.04;0.09]	0.07	0.96
*Mdn*_RT_	0.72	0.64 (0.49;0.90)	103.22 (69)	0.00	0.06 [0.04;0.09]	0.07	0.96
***+RT Quantiles***							
Q_0.20_	0.70	0.63 (0.39;0.90)	124.12 (69)	0.00	0.08 [0.06;0.10]	0.08	0.94
Q_0.40_	0.72	0.64 (0.48;0.90)	115.20 (69)	0.00	0.07 [0.05;0.10]	0.07	0.95
Q_0.60_	0.72	0.64 (0.49;0.91)	109.82 (69)	0.00	0.07 [0.04;0.09]	0.07	0.96
Q_0.80_	0.72	0.63 (0.48;0.90)	99.92 (69)	0.01	0.06 [0.03;0.08]	0.06	0.97
Q_1.00_	0.62	0.54 (0.27;0.91)	94.39 (69)	0.02	0.05 [0.02;0.08]	0.08	0.97
***+RT Variability***							
*SD*_RT_	0.57	0.51 (0.28;0.89)	99.21 (69)	0.01	0.06 [0.03;0.08]	0.09	0.96
log(*V*_RT_)	0.58	0.51 (0.27;0.89)	96.17 (69)	0.02	0.06 [0.02;0.08]	0.09	0.97
IQR	0.57	0.46 (0.20;0.75)	110.24 (69)	0.00	0.07 [0.04;0.09]	0.08	0.93
***+Errors***							
Error Rate	0.78	0.63 (0.41;0.75)	110.16 (69)	0.00	0.07 [0.04;0.09]	0.07	0.93
Probit (Error)	0.74	0.59 (0.40;0.70)	104.59 (69)	0.00	0.06 [0.04;0.09]	0.08	0.93

RMSEA with 95% CI; ω = McDonald’s omega.

**Table A2:** Measurement models and model fit of mental speed task-specific factor models.

Model	Measurement Model		Model Fit				
	ω	Mean Loadings (Range)	χ^2^ (*df)*	*p*	RMSEA [CI]	SRMR	CFI
***Search Tasks***							
***Speed***							
1/RT	0.85	0.81 (0.74;0.85)	25.98 (18)	0.1	0.06 [0.00;0.11]	0.05	0.97
***Response Time***							
*M*_RT_	0.87	0.83 (0.77;0.87)	25.05 (18)	0.12	0.06 [0.00;0.10]	0.05	0.98
*M*_log(RT)_	0.86	0.82 (0.76;0.86)	25.50 (18)	0.11	0.06 [0.00;0.10]	0.05	0.97
*Mdn*_RT_	0.83	0.79 (0.76;0.82)	27.16 (18)	0.08	0.06 [0.00;0.11]	0.05	0.97
***RT Quartiles***							
Q_0.20_	0.81	0.77 (0.70;0.81)	36.83 (18)	0.01	0.09 [0.05;0.13]	0.05	0.93
Q_0.40_	0.82	0.78 (0.72;0.81)	29.90 (18)	0.04	0.07 [0.02;0.12]	0.05	0.95
Q_0.60_	0.84	0.80 (0.78;0.81)	29.25 (18)	0.05	0.07 [0.01;0.12]	0.05	0.96
Q_0.80_	0.87	0.84 (0.80;0.86)	23.54 (18)	0.17	0.05 [0.00;0.10]	0.04	0.98
Q_1.00_	0.8	0.75 (0.66;0.80)	18.41 (18)	0.43	0.01 [0.00;0.08]	0.05	1
***RT Variability***							
*SD*_RT_	0.86	0.82 (0.76;0.91)	19.51 (18)	0.36	0.03 [0.00;0.09]	0.04	1
log(*V*_RT_)	0.86	0.82 (0.77;0.91)	18.01 (18)	0.45	0.00 [0.00;0.08]	0.04	1
IQR	0.79	0.74 (0.63;0.86)	21.25 (18)	0.27	0.04 [0.00;0.09]	0.04	0.99
***Errors***							
Error Rate	0.76	0.72 (0.70;0.73)	25.14 (18)	0.12	0.06 [0.00;0.10]	0.05	0.96
Probit (Error)	0.78	0.73 (0.68;0.77)	20.36 (18)	0.31	0.03 [0.00;0.09]	0.05	0.99
***Comparison Tasks***							
***Speed***							
1/RT	0.92	0.89 (0.77;1.02)	20.92 (18)	0.28	0.04 [0.00;0.09]	0.04	0.99
***Response Time***							
*M*_RT_	0.92	0.88 (0.75;1.02)	18.35 (18)	0.43	0.01 [0.00;0.08]	0.04	1
*M*_log(RT)_	0.92	0.88 (0.76;1.02)	19.83 (18)	0.34	0.03 [0.00;0.09]	0.04	1
*Mdn*_RT_	0.91	0.88 (0.74;1.02)	18.41 (18)	0.43	0.01 [0.00;0.08]	0.04	1
***RT Quartiles***							
Q_0.20_	0.91	0.87 (0.76;1.00)	18.33 (18)	0.43	0.01 [0.00;0.08]	0.04	1
Q_0.40_	0.9	0.87 (0.73;1.02)	17.86 (18)	0.47	0.00 [0.00;0.08]	0.04	1
Q_0.60_	0.91	0.87 (0.73;1.01)	19.29 (18)	0.37	0.02 [0.00;0.08]	0.04	1
Q_0.80_	0.9	0.86 (0.71;1.02)	22.44 (18)	0.21	0.04 [0.00;0.10]	0.05	0.99
Q_1.00_	0.9	0.87 (0.79;0.93)	14.77 (18)	0.68	0.00 [0.00;0.06]	0.04	1
***RT Variability***							
*SD*_RT_	0.9	0.87 (0.82;0.95)	16.71 (18)	0.54	0.00 [0.00;0.07]	0.05	1
log(*V*_RT_)	0.91	0.87 (0.82;0.96)	18.14 (18)	0.45	0.01 [0.00;0.08]	0.05	1
IQR	0.85	0.80 (0.67;0.94)	21.37 (18)	0.26	0.04 [0.00;0.09]	0.05	0.99
***Errors***							
Error Rate	0.83	0.78 (0.72;0.90)	21.16 (18)	0.27	0.04 [0.00;0.09]	0.06	0.99
Probit (Error)	0.8	0.75 (0.66;0.88)	21.46 (18)	0.26	0.04 [0.00;0.09]	0.06	0.98
***Substitution Tasks***							
***Speed***							
1/RT	0.92	0.89 (0.85;0.91)	25.63 (18)	0.11	0.06 [0.00;0.11]	0.04	0.98
***Response Time***							
*M*_RT_	0.92	0.89 (0.86;0.91)	27.79 (18)	0.07	0.07 [0.00;0.11]	0.04	0.98
*M*_log(RT)_	0.92	0.89 (0.86;0.91)	27.05 (18)	0.08	0.06 [0.00;0.11]	0.04	0.98
*Mdn*_RT_	0.9	0.87 (0.83;0.90)	27.74 (18)	0.07	0.07 [0.00;0.11]	0.04	0.98
***RT Quartiles***							
Q_0.20_	0.92	0.89 (0.88;0.91)	30.44 (18)	0.03	0.07 [0.02;0.12]	0.04	0.97
Q_0.40_	0.91	0.88 (0.85;0.92)	32.72 (18)	0.02	0.08 [0.03;0.12]	0.04	0.97
Q_0.60_	0.9	0.87 (0.84;0.90)	26.57 (18)	0.09	0.06 [0.00;0.11]	0.04	0.98
Q_0.80_	0.89	0.86 (0.83;0.89)	19.04 (18)	0.39	0.02 [0.00;0.08]	0.03	1
Q_1.00_	0.91	0.88 (0.83;0.93)	36.25 (18)	0.01	0.09 [0.05;0.13]	0.04	0.96
***RT Variability***							
*SD*_RT_	0.88	0.84 (0.79;0.90)	18.46 (18)	0.43	0.01 [0.00;0.08]	0.03	1
log(*V*_RT_)	0.87	0.83 (0.80;0.90)	19.10 (18)	0.39	0.02 [0.00;0.08]	0.04	1
IQR	0.78	0.74 (0.71;0.76)	17.18 (18)	0.51	0.00 [0.00;0.08]	0.04	1
***Errors***							
Error Rate	0.81	0.77 (0.74;0.81)	21.08 (18)	0.28	0.04 [0.00;0.09]	0.05	0.99
Probit (Error)	0.77	0.73 (0.66;0.78)	14.84 (18)	0.67	0.00 [0.00;0.06]	0.04	1

RMSEA with 95% CI; ω = McDonald’s omega.

**Table A3:** Measurement models and model fits of RT parameter models.

Model/Task	Measurement Model/Parameter			Model Fit				
	Loadings (Range)			χ^2^ (*df)*	*p*	RMSEA [CI]	SRMR	CFI
***Regression Models***								
***Ex-Gaussian Model***	***μ***	***σ***	***τ***					
Search	0.69 (0.64;0.75)	0.48 (0.20;0.73)	0.66 (0.60;0.69)	117.64 (59)	0.00	0.09 [0.07;0.11]	0.12	0.92
Comparison	0.82 (0.65;1.00)	0.66 (0.50;0.77)	0.80 (0.70;0.88)	63.09 (59)	0.33	0.02 [0.00;0.06]	0.05	1.00
Substitution	0.85 (0.84;0.86)	0.62 (0.54;0.70)	0.76 (0.75;0.77)	87.45 (59)	0.01	0.06 [0.03;0.09]	0.07	0.96
***Diffusion Model***	***a***	***ν***	***T_er_***					
Search	0.78 (0.69;0.88)	0.74 (0.70;0.78)	0.70 (0.64;0.80)	127.29 (59)	0.00	0.10 [0.07;0.12]	0.08	0.89
Comparison	0.84 (0.78;0.92)	0.72 (0.56;0.87)	0.77 (0.63;1.02)	73.05 (59)	0.10	0.04 [0.00;0.07]	0.06	0.98
***Correlation Models***								
***Ex-Gaussian Model***	***μ***	***σ***	***τ***					
Search	0.69 (0.64;0.75)	0.48 (0.20;0.73)	0.66 (0.60;0.69)	117.64 (59)	0.00	0.09 [0.07;0.11]	0.12	0.92
Comparison	0.82 (0.65;1.00)	0.66 (0.50;0.77)	0.80 (0.70;0.88)	63.09 (59)	0.33	0.02 [0.00;0.06]	0.05	1.00
Substitution	0.85 (0.84;0.86)	0.62 (0.54;0.70)	0.76 (0.75;0.77)	87.45 (59)	0.01	0.06 [0.03;0.09]	0.07	0.96
***Diffusion Model***	***a***	***ν***	***T_er_***					
Search	0.78 (0.69;0.88)	0.74 (0.70;0.78)	0.70 (0.64;0.80)	127.29 (59)	0.00	0.10 [0.07;0.12]	0.08	0.89
Comparison	0.84 (0.78;0.92)	0.72 (0.56;0.87)	0.77 (0.63;1.02)	73.05 (59)	0.10	0.04 [0.00;0.07]	0.06	0.98

RMSEA with 95% CI.
